# Supplementary material for: The Differential Prognostic Impact of Long-Duration Atrial High-Rate Episodes Detected by Cardiac Implantable Electronic Devices between Patients with and without a History of Atrial Fibrillation
Source: J Clin Med. 2022 Mar 21;11(6):1732. doi: 10.3390/jcm11061732 (PMC8954400; doi:10.3390/jcm11061732)
Supplement: Supplementary file 1 [file jcm-11-01732-s001.zip › jcm-1598339-supplementary.pdf]

Supplementary Materials

Table S1. Characteristics of patients who developed MACE in the group without a history of AF.

Patient No.	Age (years)	Sex	AHRE duration	Type of CIEDs	The first MACE		All-cause death		HFH		Stroke
					Type	Year	Y/N	Cause of death	Y/N	Multiple HFH	Y/N
1	54	M	≥24 h	CRTD	HFH	3.7	Y	HF	Y	N	N
2	67	M	≥24 h	ICD	HFH	8.4	N		Y	N	N
3	60	M	≥24 h	CRTD	HFH	3.4	Y	HF	Y	Y	N
4	81	F	≥24 h	CRTP	HFH	8.2	N		Y	N	N
5	52	M	≥24 h	ICD	HFH	1.9	N		Y	Y	N
6	61	M	≥24 h	ICD	HFH	5.5	N		Y	N	N
7	64	F	≥24 h	CRTD	HFH	5.9	N		Y	N	N
8	85	M	≥24 h	PM	death	8.8	Y	pneumonia	N	-	N
9	80	M	≥24 h	PM	death	8.0	Y	sudden death	N	-	N
10	69	M	≥24 h	ICD	death	9.5	Y	sudden death	N	-	N
11	74	M	≥24 h	PM	stroke	2.4	N		N	-	Y
12	45	M	≥24 h	ICD	HFH	2.7	N		Y	N	N
13	57	M	≥24 h	ICD	HFH	6.9	N		Y	N	N
14	73	M	≥24 h	ICD	HFH	0.9	N		Y	Y	N
15	84	F	≥24 h	PM	HFH	2.9	N		Y	N	N
16	75	M	≥24 h	ICD	HFH	1.4	Y	HF	Y	Y	N
17	85	M	<24 h	PM	HFH	6.1	Y	pneumonia	Y	N	N
18	82	M	<24 h	PM	death	3.2	Y	pneumonia	N	-	N
19	62	M	<24 h	CRTD	death	2.3	Y	pneumonia	N	-	N
20	54	M	<24 h	ICD	death	7.0	Y	sudden death	N	-	N
21	100	M	<24 h	PM	death	1.0	Y	senility	N	-	N

22	76	M	<24 h	ICD	stroke	2.0	N		N	-	Y
23	65	M	<24 h	CRTD	death	1.6	Y	sudden death	N	-	N
24	85	F	<24 h	PM	death	0.9	Y	GI bleeding	N	-	N

AF, atrial fibrillation; AHRE, atrial high rate episodes; CIEDs, cardiac implantable electronic devices; CRTD, cardiac resynchronization therapy with defibrillator; CRTP, cardiac resynchronization therapy with pacemaker; F, female; GI, gastrointestinal; HF, heart failure; HFH, heart failure hospitalization; ICD, implantable cardioverter defibrillator; M, male; MACE, major adverse cardiovascular events; N, no; PM, pacemaker; Y, yes
